# Robust magnetic moments on the basal plane of the graphene sheet effectively induced by OH groups

**DOI:** 10.1038/srep08448

**Published:** 2015-02-13

**Authors:** Tao Tang, Nujiang Tang, Yongping Zheng, Xiangang Wan, Yuan Liu, Fuchi Liu, Qinghua Xu, Youwei Du

**Affiliations:** 1 Physics Department & Nanjing National Laboratory of Microstructures, Nanjing University, Nanjing 210093, PR China; 2 School of Science, Guilin University of Technology, Guilin 541004, PR China

## Abstract

Inducing robust magnetic moments on the basal plane of the graphene sheet is very difficult, and is one of the greatest challenges in the study of physical chemistry of graphene materials. Theoretical studies predicted that introduction of a kind of *sp*^3^-type defects formed by OH groups is an effective pathway to achieve this goal [Boukhvalov, D. W. & Katsnelson, M. I. *ACS Nano* 5, 2440–2446 (2011)]. Here we demonstrate that OH groups can efficiently induce robust magnetic moments on the basal plane of the graphene sheet. We show that the inducing efficiency can reach as high as 217 *μ*_B_ per 1000 OH groups. More interestingly, the magnetic moments are robust and can survive even at 900°C. Our findings highlight the importance of OH group as an effective *sp*^3^-type candidate for inducing robust magnetic moments on the basal plane of the graphene sheet.

Magnetic properties of graphene are gaining increasing interest recently for its potential application in spintronics, since its weak spin-orbit interaction and long spin-relaxation time provide ideal conditions to manipulate the spins^1^,^2^,^3^,^4^. However, direct application of graphene in magneto-electronics is unavailable because perfect graphene is intrinsically non-magnetic and absent of localized magnetic moments, owning to the π-symmetry electron system lack of uncompensated electron spins^4^,^5^. In nature, approaches to introduce magnetic moments in graphene are to break down the symmetry of its bipartite honeycomb lattice. Experimentally, creating robust magnetic moments in graphene remains very difficult, and is one of the greatest challenges in the study of the physical chemistry of graphene materials^6^. In principle, the approaches can be roughly divided into two categories: the edge-type approach (creation of the edge magnetic moments at the edge sites by edge-type defects) and the basal-plane approach (creation of the basal-plane magnetic moments on the basal-plane sites by *sp*^3^-type defects). For the edge-type approach, it mainly includes ion irradiation to create point vacancies^4^,^7^,^8^,^9^,^10^,^11^,^12^, introduction of line vacancies by formation of graphene nanomesh^13^ or synthesis of graphene nanoribbon^12^,^14^,^15^,^16^, or adsorption of heteroatoms such as nitrogen at the edge sites of the graphene sheet^17^, *etc*. The biggest weakness of this approach is that it can only induce limited edge magnetism at the vacancy or edge sites of the graphene sheet. For example, although a single isolated vacancy can theoretically induce magnetic moment *μ* ~ *μ*_B_^4^, the total vacancy density is restricted to a limited value to maintain the integrity of the graphene sheet and, hence the density of magnetic moments is very low^8^. And what is worse, the edge magnetic moments are unstable because they are fragile to be passivated by external surroundings^9^. Notably, the edge magnetic moments generated by N adatoms are stable because of the high stability of N adatoms. However, because N adatoms are usually chemisorbed on the vacancy or edge sites, the density of N-induced magnetic moments is again low^17^.

Quite differently from the edge-type approach to create defects only at vacancy or edge sites, the basal-plane approach is to introduce *sp*^3^-type defects on the basal plane of the graphene sheet and, thus more defects can be introduced. Therefore, higher spin density is expected by this approach, and which is considered as hitherto the most promising approach to induce robust magnetic moments on the graphene sheet^8^. This approach has been realized by F or H adatoms^8^,^18^,^19^,^20^,^21^,^22^,^23^. However, such adatoms tend to aggregate arising from the low migration barrier, so that the inducing efficiency of magnetic moments is quite low^5^,^6^,^8^,^18^,^19^,^20^,^24^,^25^,^26^. Additionally, both hydrogenated and fluorinated graphene are easy to lose their adatoms at moderate temperatures (hydrogenated graphene, ~200°C and the fluorinated one, ~400°C)^25^,^26^ and consequently, the magnetic graphene will convert back into the non-magnetic one with the recovery of the aromatic structure. Therefore, it is important to search another *sp*^3^-type alternative that can effectively induce robust magnetic moments on the basal plane of the graphene sheet.

Recently, theoretical studies confirmed that the chemisorption of a single OH group on the basal plane of graphene can introduce magnetic moment ~1 *μ*_B_^6^,^27^,^28^. But the great difference is, not like F or H adatoms clustering^6^,^8^,^20^, OH groups on graphene sheet can be stable because of the suitable binding energy and relatively high migration barrier of C–OH bond^6^,^29^,^30^,^31^. More importantly, the stability can be strengthened by the emergence of ripples and surface topology^6^,^32^,^33^ and/or the coincidence of symmetry of the clusters with the graphene lattice^6^, suggesting the high feasibility of creating robust magnetic moments by OH groups. Furthermore, it has been demonstrated that OH group is far more thermally stable even surviving at 1000°C^34^,^35^,^36^,^37^. Notably, given so much superiority of OH group to induce magnetic moments in graphene, the relevant experimental study is still scarce.

In this study, we prepared hydroxylated graphene (OHG) with a very high magnetization of 2.41 emu/g by annealing of graphene oxide (GO) to remove the unstable oxygen groups and leave the stable OH groups. We demonstrate that OH groups can effectively induce robust magnetic moments on the basal plane of the graphene sheet. Our results highlight the two great superiorities of OH groups: high magnetic inducing efficiency (217 *μ*_B_ per 1000 OH groups) and high stability (surviving even at 900°C). Our results demonstrate that OH group is an effective candidate for inducing robust magnetic moments on the basal plane of the graphene sheet. These results have important implications for the synthesis of high-magnetization graphene for future applications of spintronics. In particular, the robust magnetic moments we observe in OHG provide the prerequisite to induce long-range magnetic ordering. Conversely, the ferromagnetic coupling between these basal-plane magnetic moments still needs to be addressed for the realization of ferromagnetic graphene with high magnetization.

## Results

### Synthesis and characterization of aGO and the OHG samples

It has been generally accepted that (i) oxygen groups present on GO mainly in forms of epoxy, OH, carbonyl, and carboxyl groups; and (ii) most epoxy and OH groups lie on the basal plane of the graphene sheet, and carbonyl and carboxyl groups can only sit at the edge or vacancy sites^38^,^39^. Prior to any discussions about GO, we should mention that previous experimental results of the magnetic properties of GO via chemical exfoliation of graphite are multifarious^15^,^16^, which may result from the great differences in the content, type, and distribution of oxygen groups depending on the starting graphite material and oxidative conditions^40^,^41^. We performed systematical experiments and found that the lightly oxidized GO has high OH content and high magnetization (see Ref. 42 and Supplementary Fig. S1). By reducing the oxidation duration^40^,^42^,^43^, we prepared the as-prepared GO (aGO) with high magnetization.

It is known that (i) epoxy is nonmagnetic for it creates equal defects in A and B sublattice^4^, by contrast, a single OH group can induce ~1 *μ*_B_ on the basal plane of the graphene sheet^6^,^27^,^28^; and (ii) epoxy is thermally unstable and can be easily removed by annealing, leaving only stable OH groups on the basal-plane sites^34^,^35^,^36^,^37^. Therefore, to increase the magnetization of aGO for clarifying the magnetic contribution of OH groups, we synthesized the OHG samples by annealing aGO at different temperatures. We divide the OHG samples into two groups: Group A (including OHG-200, OHG-300, OHG-400, and OHG-500) and Group B (including OHG-600, OHG-700, OHG-800, and OHG-900) (numbers indicate the annealing temperatures). We note that annealing aGO at 950°C, there was no obviously substantial residue.

**Figure 1:**
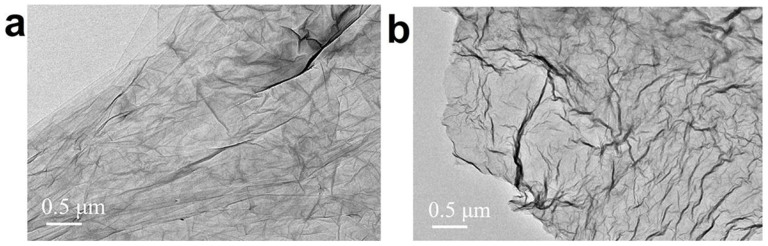
Typical TEM images. (a) aGO and (b) OHG-600.

The atomic force microscope (AFM) image of aGO shows that the heights of most of them are ~0.7 nm (see Supplementary Fig. S2a), indicating that most of the sheets are monolayered. The Raman spectra (see Supplementary Fig. S2b) also confirmed it. Shown in Figs. 1a and 1b are the typical transmission electron microscope (TEM) images of aGO and OHG-600. One can find that both aGO and OHG-600 maintain two-dimensional ultrathin flexible structure and μm scale integrity of the sheets, in addition to the emergence of more ripples and scrollings in OHG-600.

**Figure 2:**
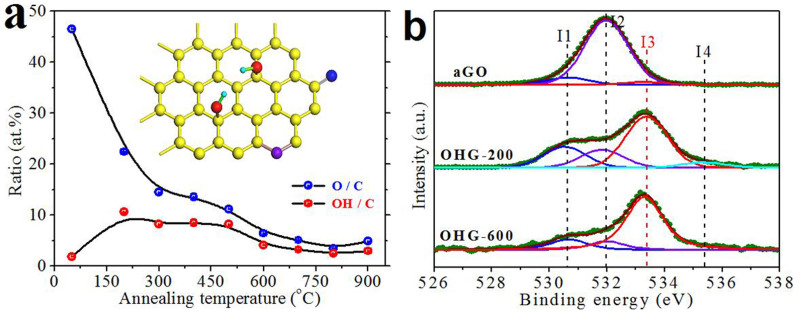
O content and its bonding environments of aGO and the OHG samples obtained at different annealing temperatures. (a) Dependences of oxygen to carbon ratio (O/C, blue dots) and OH coverage (OH/C, red dots) measured or calculated according to the XPS spectra on the annealing temperature. The lines shown are guides to the eye only. Inset, schematic representation of typical oxygen groups in OHG sheet. Red, purple, and blue balls denote OH-type, ether-type and carbonyl-type O atoms, respectively. C atoms are yellow and H atoms are cyan. (b) Typical fine-scanned XPS spectra of O 1 s. The symbols specify the following groups: I1, I2, I3, and I4 represent carbonyl, epoxy and/or ether, OH, and chemisorbed or intercalated adsorbed water, respectively.

To detect the O contents and the bonding environments of aGO and the OHG samples, X-ray photoemission spectroscopy (XPS) measurements were carried out. As shown in Fig. 2a, it is found that (i) after annealing at 300°C, the O/C ratio sharply decreases from ~46.54 to 14.47 at.%; and (ii) with further increase of the annealing temperature, it keeps a stable value ~5 at.%, indicating that a portion of C–O bondings are thermally stable. Figure 2b shows the typical fine-scanned O 1 s spectra of aGO and the OHG samples. To make clear which kinds of these stable bondings are, we carefully performed the deconvolution of the fine-scanned O 1 s spectra. Considering the fact that deconvolution of the fine-scanned XPS spectra is inevitably more or less subjective, we tried to make it accurate by strictly constraining each component peak fixed at the same full width at half maximum (FWHM) and fitting to Voigt functions having 70% Gaussian and 30% Lorentzian character after performing a Shirley background subtraction. The three main peaks of O 1 s spectra around 530.88 ± 0.2, 531.83 ± 0.2, and 533.38 ± 0.2 eV were assigned to carbonyl (>C = O), epoxy/ether (aliphatic C–O), and OH (hydroxyl C–O), respectively^34^,^44^. One can find that (i) the predominant bonding in aGO is epoxy/ether, and (ii) after annealing, epoxides were removed and only small fraction of thermally stable ether rings left resident at the edges or vacancies of OHG sheets (see inset of Fig. 2a)^34^,^45^. Just like ethers, carbonyl groups, which also can only lie at edge or vacancy sites, are hard to be completely removed (see inset of Fig. 2a). For OH groups, things are completely different. OH groups can covalently bond to the basal-plane carbon atoms to form *sp*^3^-type defects (see inset of Fig. 2a), not only limited to edge or vacancy sites. Therefore, the content of OH groups can be much higher than those of thermally stable ether and/or carbonyl groups in OHG. As shown, the peak of OH groups in aGO is relatively weak, but after annealing it obviously prevails over ether and carbonyl groups. By calculating the peak area ratios, we get the OH group ratios in oxygen bondings, which are ~4.0%, 47.3%, 56.6%, 62.6%, 73.2%, 63.4%, 62.5%, 72.6%, and 59.7% for aGO, OHG-200, OHG-300, OHG-400, OHG-500, OHG-600, OHG-700, OHG-800, and OHG-900, respectively. Note that, although the O contents of Group B samples are relatively low and the O 1 s spectra fluctuate somewhat, one can still clearly see that the peak of OH groups is the predominant one (see Fig. 2b and Supplementary Fig. S3). Furthermore, the C 1 s fine-scanned spectra of the OHG samples (see Supplementary Fig. S4) demonstrate further that OH groups are the uppermost component of oxygen-containing groups. The peak around 535.34 eV corresponds to chemisorbed/intercalated water molecules^34^. The signal of H_2_O in aGO is inconspicuous, which may attribute to that the signal of H_2_O is flooded by the enormous epoxy/ether signal.

According to the O contents and C–OH ratios in oxygen bondings, we calculated the OH coverage (OH/C) of aGO and the OHG samples (Fig. 2a). The OH coverage of aGO is only 1.8 at.%. Interestingly, it increases as high as 10.6 at.% for OHG-200. It should be noted that by using 51.0 mg aGO annealed at 200°C for an hour, we obtained 21.6 mg OHG-200. Combined with the O/C atomic ratio measured by XPS, we know that the weight loss of carbon is *ca.* 34%. Therefore, one can conclude that many new OH groups were created with the decomposition of other unstable oxygen groups during annealing^34^. As shown in Fig. 2a, it is found that (i) OH group, which is the only one which can form *sp*^3^-type defect, is predominant among all the oxygen groups in all the OHG samples, and (ii) the dominance increases with the increase of the annealing temperature. Group A samples have a high OH coverage ~8 at.%, while in Group B, the coverage decreases to a steady value ~3 at.%, implying that a number of OH groups decomposed at high temperatures. Even so, the OH coverage of OHG-900 still maintains 2.96 at.%, confirming the high thermal stability of OH group^34^,^35^,^36^,^37^. Briefly, by annealing of aGO below 600°C to remove unstable oxygen groups and generate extra OH groups, we have prepared OHG with high OH coverage. The coverage can be tuned in a wide range from *ca*. 3 to 10 at.% by changing the annealing temperature.

### Magnetic properties of aGO and the OHG samples

**Figure 3:**
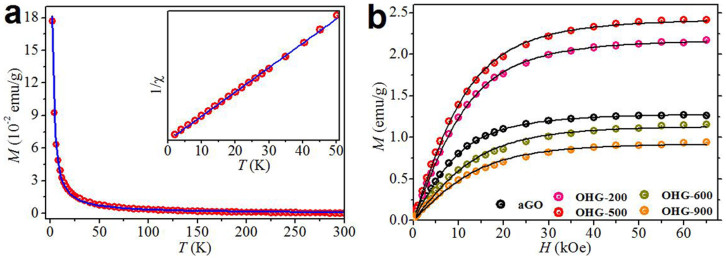
Magnetic properties of aGO and the OHG samples obtained at different annealing temperatures measured by a superconducting quantum interference device (SQUID) magnetometer. (a) Typical mass magnetization dependence on the temperature (*M – T*) of OHG-500 measured from 2 to 300 K under the applied field *H* = 1 kOe. Inset is the corresponding 1/*χ – T* curve. Red symbols are the measurements and black solid lines are fitted by the Curie law. (b) Mass magnetization dependences on the applied magnetic field (*M – H*) of aGO and the OHG samples measured at 2 K. Colorful symbols are the measurements and solid lines are fit to Brillouin function with *g* = 2.

Figure 3a shows the typical dependence of mass magnetization *M* of OHG-500 on temperature *T* and it fits well with Curie law *χ = C/T. χ* is the susceptibility and *χ = M/H. H* is the applied magnetic field. Inset is the corresponding 1/*χ – T* curve, which demonstrates a linearly, purely Curie-like paramagnetic behavior. The *M – T* dependences of aGO and other OHG samples were also measured, and they are all Curie-like paramagnetic just like OHG-500. The *M – H* dependences were also measured. At 300 K, all the samples are linearly diamagnetic (see Supplementary Fig. S5a). In contrast, at 2 K, all the samples are strongly paramagnetic (Fig. 3b), indicating high-density localized magnetic moments in them. The *M – H* curves are well fitted using the Brillouin function
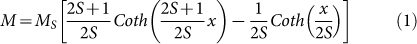
where the saturated magnetization *M_s_* = *NgSμ_B_*, *x* = *gSμ_B_H*/(*k_B_T*), *k_B_* is the Boltzmann constant, *N* is the number of present magnetic moments, *S* is the spin angular momentum number, and *g* is the Landau factor assumed to be 2. The fitted values for all the samples are summarized in Table 1. It is found that aGO has a high *M_s_* of 1.27 emu/g, much higher than the value observed in GO with long oxidation duration (see Supplementary Fig. S1). Interestingly, *M_s_* increases with annealing temperature from 200 to 500°C and then decreases with further increasing the annealing temperature to 900°C. The maximum is 2.41 emu/g for OHG-500. We recall the previous reports about the magnetization of graphene and its derivatives, and find that *M_s_* of OHG-500 is much higher than the values reported (see Supplementary Table S1).

We should note that graphene is highly light-weight and the measured magnetic signal of milligram-scale graphene sample is generally very weak. Thus, the contamination with very small volume fraction of 3 d impurities can result in large deviation in the intrinsic *M_s_* of graphene. To minimize the deviation, we compressed and loaded dozens of milligram sample for measurement each run. As shown in the Supplementary Fig. S5b, the 2 K magnetic signal of aGO is as high as 0.061 emu, which is very robust and clear. After measured the magnetic properties of all the samples, we carefully performed inductively coupled plasma (ICP) spectrometric measurements and completely excluded the magnetic contribution of 3 d impurities (see Supplementary Table S2).

Moreover, also by fitting the corresponding *M – H* curves, one can find that the *S* value of aGO is very close to the quantum number 5/2, corresponding to 5 spins ferromagnetically coupled together. It is greatly different from the cases of only small magnetic moment of 1 *μ*_B_ observed in ion-irradiated and fluorinated graphene^8^,^20^. Note that the large magnetic moment of 5 *μ*_B_ was previously observed in ultrasonic exfoliated graphene laminates^29^, and was considered to be caused by (OH)_7_ groups^6^. These facts imply that the emergence of the large magnetic moment of 5 *μ*_B_ in our aGO is not occasional, and which is further confirmed by our repeated independent experiments (see Ref. 42 and Supplementary Fig. S1a). While in the case of the OHG samples, *S* deviate from spin 5/2 state to a certain extent. For example, Group A samples have *S* value of about 2, and which degenerates further to about 1.7 for Group B samples. Clearly, annealing results in the degeneration of the high-spin state.

## Discussion

We now consider the magnetic source for the robust magnetic moments observed in aGO and the OHG samples. As mentioned above, our OHG is a kind of defective graphene with *ca*. 3–10 at.% OH coverage, in addition to few other oxygen groups (carbonyl or ether) located at the vacancies and/or edges. Generally speaking, some factors, such as isolated vacancies^8^,^11^ and zigzag edges^14^,^15^, *etc*, may only contribute to the edge magnetism. However, the vacancies and edges generated by annealing GO are disordered^46^, and dangling bonds terminated by oxygen groups (such as carbonyl or ether)^6^,^47^, the unavoidable emergence of double vacancies, Stone-Wales defects, and so on, would lead to nonmagnetic states reconstruction^12^. Based on the theoretical predictions that OH groups can induce robust magnetic moments on the basal plane of graphene^6^,^27^,^28^,^31^ and our experimental evidence of very high intrinsic magnetization, one can conclude that OH groups are the mainly magnetic source of aGO and the OHG samples.

**Figure 4:**
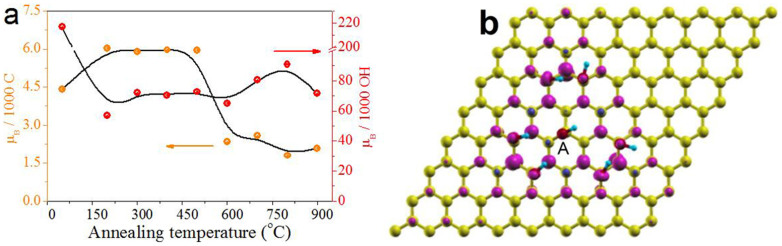
Robust magnetic moments induced by OH groups on the basal plane of graphene sheet. (a) Dependences of spin density (orange circles) and the magnetic inducing efficiency of OH groups (red circles) on the annealing temperature. The solid lines are the guides for the eye only. (b) Schematic illustration of (OH)_7_ cluster on the basal plane of the graphene sheet and the corresponding spin charge density distribution with contour spacings 1 × 10^−2^ e/Å^3^. Carbon, oxygen, and hydrogen atoms are yellow, red, and cyan, respectively. Capital A denotes the OH group in (OH)_7_ cluster which sits on sublattice A of graphene sheet. Majority and minority spins are purple and blue, respectively.

To take a deeper insight into the magnetic properties of aGO and the OHG samples, we calculated their spin densities (Fig. 4a) according to the C contents and fitted *M_s_*. The C contents are calculated based on the XPS results of C and O atomic ratio. In short, if the O/C atomic ratio of the sample is *x* and the OH coverage is *y*, its chemical formula can be represented as CO*_x_*H*_y_*. It is found that the spin density is ~4.42 *μ*_B_ per 1000 C for aGO, which increases up to ~6 *μ*_B_ per 1000 C for the OHG samples of Group A. In view of the fact that the weight loss of aGO by annealing aGO at 200°C is *ca.* 34%, one can make a reasonable assumption that magnetic contribution of the newly annealing-created OH groups is low. The reason may be that the newly created OH groups prefer to lie at edges or vacancies^34^, and the created OH-passivated edges or vacancies are generally nonmagnetic or low-spin state^6^,^9^. Moreover, some of the new OH groups also may lie on the basal plane of the graphene sheet. However, because of the removal of oxygen groups by annealing, the newly OH groups may form large clusters, resulting in that the total magnetic inducing efficiency is low. In other words, it just suggests that the magnetic moments of OHG are mainly contributed by the original basal-plane OH groups. For Group B samples, the spin density decreases to ~2 *μ*_B_ per 1000 C, this may result from both the decrease of the OH coverage (Fig. 2a) and the clustering of OH groups at a high annealing temperature. According to the OH coverage, we calculate the magnetic inducing efficiency of OH group (Fig. 4a). Surprisingly, it is found that inducing efficiency is very high of ~217 *μ*_B_ per 1000 OH groups for aGO, two orders of magnitude higher than those of other *sp*^3^-type counterparts reported^8^,^18^,^20^. It decreases to ~60 *μ*_B_ per 1000 OH groups for OHG-200, further suggesting that the magnetic moments of OHG are mainly induced by the original OH groups rather than the newly annealing-generated. It's worth highlighting that OHG-900 still has such a high spin density (2.08 *μ*_B_ per 1000 C) and inducing efficiency (71.5 *μ*_B_ per 1000 OH groups). It indicates that the magnetic moments are robust despite that some of them vanished with the unavoidable decomposition of original ‘magnetic’ OH groups on the basal plane of the OHG sheet.

The robust magnetic moments induced by the basal-plane OH groups on aGO and OHG sheets can be explained by a number of possible mechanisms. It is considered that the migration of OH groups during the delamination of graphite can lead to the formation of (OH)_7_ clusters on the basal plane of the graphene sheet^47^,^48^. Boukhvalov and Katsnelson have theoretically predicted that such an (OH)_7_ cluster can induce a large magnetic moment of 5 *μ*_B_^6^. Based on their computational results^6^ and Lieb's theorem^49^, a possible (OH)_7_ cluster configuration inducing 5 *μ*_B_ was sketched in Fig. 4b: one OH group is chemisorbed by a carbon atom from sublattice A of graphene and six other OH groups sit at the second neighbors belonging to sublattice B. Our density functional theory (DFT) calculation revealed that this configuration has a total magnetic moment of 4.91 *μ_B_* (Fig. 4b), close to the experimental and theoretical results^6^,^29^,^42^. It suggests that this (OH)_7_ cluster may be the magnetic source of the large magnetic moments observed in our samples. Generally, because of the low migration barrier of adatoms clustering^6^,^8^,^30^, the induced large magnetic moments in graphene are metastable. However, if the distance between the (OH)_7_ clusters are greater than 3 nm from one another on the flat graphene sheet, the reconstruction barrier can be *ca.* 0.4 eV, and the stability could be further enhanced by the emergence of ripples and surface topology^6^,^32^,^33^ and/or by the coincidence of symmetry of the clusters with the graphene lattice^6^. Actually, we have observed lots of ripples in both aGO and OHG sheets (Figs. 1a and 1b). What's more, the oxygen groups, such as the high-density epoxy on the basal plane of aGO sheets and ether groups at vacancy sites in OHG sheets (Figs. 2a and 2b), will also hinder the migration of (OH)_7_ clusters^20^,^39^.

Inducing high-density magnetic moments on graphene is usually the prerequisite to induce long-range magnetic ordering^10^, therefore, OHG would be a more competitive alternative to realize it. Even so, no obvious ferromagnetic or antiferromagnetic behavior was detected in our samples. According to the spin density, we can speculate the average distance between the adjacent large magnetic moments in OHG. For instance, in Group A samples, ~1.5 magnetic cluster with *S* = 2 are generated by 1000 C atoms, so on average, a 25 × 25 or 26 × 26 graphene supercell (~666 C atoms) contributes a large magnetic moment. Assuming that the graphene sheet is flat and the magnetic moments distribute uniformly, we obtain that the average distance is at least 6 nm. Since the OH group interaction is negligible when the space is larger than 3 nm^6^, such a space is large for the notable inter-cluster interaction could work. Apparently, the large space ensures the magnetic moments independently stably exist, but at the same time, the too weak inter-cluster exchange interaction restricts the formation of long-range magnetic ordering. It may be the reason why no obvious long-range magnetic ordering was detected in all of our samples even though they have such high densities of magnetic moments.

In addition, we should note that even the highest efficiency observed in aGO is as high as 217 *μ*_B_ per 1000 OH groups, it is still lower than the theoretical value of 1 *μ*_B_ per single OH group. Namely, most of the OH groups on the aGO and OHG sheets do not contribute magnetic moments. It may attribute to the analogous clustering of OH groups, similar to the case of fluorine on graphene sheet^8^,^20^. To realize long-range magnetic ordering in OHG would demand those OH-group clusters arranged in a certain pattern, while so far it seems technically difficult. To shorten the adjacent space of magnetic moments, we tried to import extra OH groups^50^ for inducing extra magnetic moments on the OHG sheets. We observed the clear increase in magnetic moments in Group B samples (see Supplementary Fig. S6a), but also detected the decrease in Group A sample (see Supplementary Fig. S6b). In any case, OH-imported OHG remains purely paramagnetic. However, we believe that altering the distribution of OH groups or interaction among magnetic moments would have a significant chance to tune the magnetic properties of OHG and, thus realize long-range magnetic ordering.

In summary, we have obtained aGO with high spin density of 4.42 *μ*_B_ per 1000 C by reducing the oxidation duration, and have observed a very high magnetic inducing efficiency of 217 *μ*_B_ per 1000 OH groups. By annealing of aGO to remove the ‘non-magnetic’ unstable oxygen groups and leave the ‘magnetic’ stable OH groups, we have obtained OHG with very high magnetization of 2.41 emu/g. Our results provide the solid experimental evidence that OH groups can induce robust magnetic moments on the basal plane of the graphene sheet. More importantly, we demonstrate that the magnetic moments are highly stable and can survive even at 900°C. Our results provide an effective *sp*^3^-type functional group for generating robust magnetic moments in graphene and, thus represent an important step for the potential applications of spintronics.

## Methods

### Preparation of aGO and the OHG samples

The as-prepared GO was synthesized by chemical exfoliation of 8 g natural flake graphite powder (500 mesh)^42^. In a typical experiment, the mixture of 8 g graphite, 8 g NaNO_3_, 48 g KMnO_4_, and 384 ml condensed H_2_SO_4_ was stirred for 1.5 h at 0°C, and then followed by another 2 h stirring at 35°C. Thereafter, 320 ml H_2_O was added into the mixture within 15 min at a steady flow. Without any delay, the premix of 800 ml H_2_O and 40 ml H_2_O_2_ was added within 6 min at a steady flow. During the process, the temperature of the solution fixed at 35°C. To avoid deeper oxidation, the mixture was immediately transferred to other container and diluted with 2000 ml H_2_O. The obtained solution was washed for 13 times by repeated 30 min centrifugation at 13000 rpm. The sediment was redispersed into water and centrifugated at 6000 rpm for 20 min, and only the supernatant was left to get GO sheets with high few-layer ratio. Finally, the lightly oxidized aGO was obtained by drying the GO solution in vacuum freeze drier at 50°C. To avoid other extra source which may induce magnetic moments, no ultrasonication was adopted. The OHG samples were synthesized by annealing aGO in Ar atmosphere at different temperatures for 1 h.

### Characterization

The morphologies of the samples were investigated by TEM (model JEM–2100, Japan) and AFM (Veeco dimension V, USA). XPS measurements were performed on PHI5000 VersaProbe (ULVAC-PHI, Japan) using Al Ka radiation. Raman images were performed on inVia confocal Raman microscope (Renishaw, UK) using a laser excitation of 532 nm. The magnetic properties of the samples were measured using SQUID magnetometer with a sensitivity less than 10^−8^ emu (Quantum Design MPMS-XL, USA), and all data have been corrected for the diamagnetic contribution by subtracting the corresponding linear diamagnetic background at room temperature. The 3 d impurity elements of all the samples are measured by inductively coupled plasma (ICP) spectrometry (Jarrell-Ash, USA).

### DFT calculations

Our calculation was performed by density function theory as implemented in the SIESTA code^51^. The generalized gradient approximation (GGA-PBE)^52^ is adopted to treat electron exchange and correlation, which is considered as the most suitable approximation for the graphene-adatom system^31^. Double-ζ plus polarized basis set for local orbitals and norm-conserving Troullier-Martins for core pseudopotentials were used to simulate the systems. Optimization of the force and total energy was performed with an accuracy of 0.04 eV/Å and 0.1 meV, respectively. The calculations were carried out with an energy mesh cutoff of 360 Ry and a k-point mesh of 11 × 11 × 1 in the Mokhorst-Park schemes used for the Brillouin Zone integrations^53^. The spin-polarized mode was considered in our system. To avoid the interlayer interactions, the size of vacuum in the direction perpendicular to graphene plane in our system is set to 20 Å.

## Author Contributions

N.J.T. proposed and supervised the project, T.T. and N.J.T. designed the experiments, T.T. and Y.L. carried out the experiments, Y.P.Z. performed the DFT calculations, T.T., X.G.W. and N.J.T. analyzed the data and wrote the manuscript. Y.P.Z., X.G.W., Y.L., F.C.L., Q.H.X. and Y.W.D. had valuable discussions and edited the manuscript. All the authors participated in discussion of research.

## Supplementary Material

Supplementary Information Supplementary Information

## Figures and Tables

**Table 1:** The best fitted values of saturated magnetization *M_s_* and spin angular momentum number *S* of aGO and the OHG samples by using the Brillouin function

samples	aGO	Group A				Group B			
		OHG-200	OHG-300	OHG-400	OHG-500	OHG-600	OHG-700	OHG-800	OHG-900
*M_s_* (emu/g)	1.27	2.16	2.30	2.35	2.41	1.14	1.12	1.01	0.91
*S*	2.51	2.01	2.09	2.02	2.07	1.71	1.74	1.70	1.65
