# Supplementary material for: Robust magnetic moments on the basal plane of the graphene sheet effectively induced by OH groups
Source: Sci Rep. 2015 Feb 13;5:8448. doi: 10.1038/srep08448 (PMC4327573; doi:10.1038/srep08448)
Supplement: Supplementary Information [file srep08448-s1.doc]

**Supplementary Information**

Robust magnetic moments on the basal plane of the graphene sheet effectively induced by OH groups

Tao Tang^1,2^, Nujiang Tang^1^, Yongping Zheng^1^, Xiangang Wan^1^, Yuan Liu^1^, Fuchi Liu^1^, Qinghua Xu^1^ & Youwei Du^1^

^1^Physics Department & Nanjing National Laboratory of Microstructures, Nanjing University, Nanjing 210093, PR China. ^2^School of Science, Guilin University of Technology, Guilin 541004, PR China.

**Supplementary Figure S1 | aGO with shorter oxidation duration generally has a higher magnetization, and all the fitted *S* values are ~ 5/2.** (**a**) Mass magnetization dependences on applied magnetic field (*M – H*) of the GO samples with different oxidation duration measured at 2 K. Colorful symbols are the measurements and solid lines are fit to Brillouin function with *g* = 2. *S* is the fitted spin angular momentum number. (**b**) Typical fine-scaned C 1s XPS spectra. GO-0h (aGO in the main text), GO-8h, GO-48h, GO-200h are the samples oxidized for different hours at 35 °C after the premix of 800 ml H_2_O and 40 ml H_2_O_2_ was added (see Methods in the text). During the oxidation process, the solution was kept stirring. Other steps are exactly the same as which GO-0h was prepared. As shown in Fig. S1a，*S* values of all the GO samples are similar ~ 5/2, while the *Ms* decreased with the increase of the oxidation time. To detect the O contents and the bonding environments of these GO samples, X-ray photoemission spectroscopy (XPS) measurements were carried out. It is found that with the increase of the oxidation time, the O content increases. For instance, the atomic ratio of O to C of GO-0h is 46.3 at.% and that of GO-200h is 49.0 at.%. Moreover, with the increase of the oxidation time, the change of its bonding environment is evident. As shown in Fig. S1b, the peak stands at ~ 284.5 eV corresponding to C–C sp^2^ bonding, ~ 286.8 eV to C–O bonding (epoxy and/or OH groups on basal plane of graphene sheet), and ~ 288.5 eV to C=O (carboxyl and/or carbonyl at edge or vacancy sites of graphene sheet) peak. Combined with the magnetic results (Fig. S1b), it is found that GO with longer oxidation time generally has a higher OH content and a higher magnetization. Anyway, our repeated experiments indicate that the lightly oxidized GO has the high OH content and high magnetization.


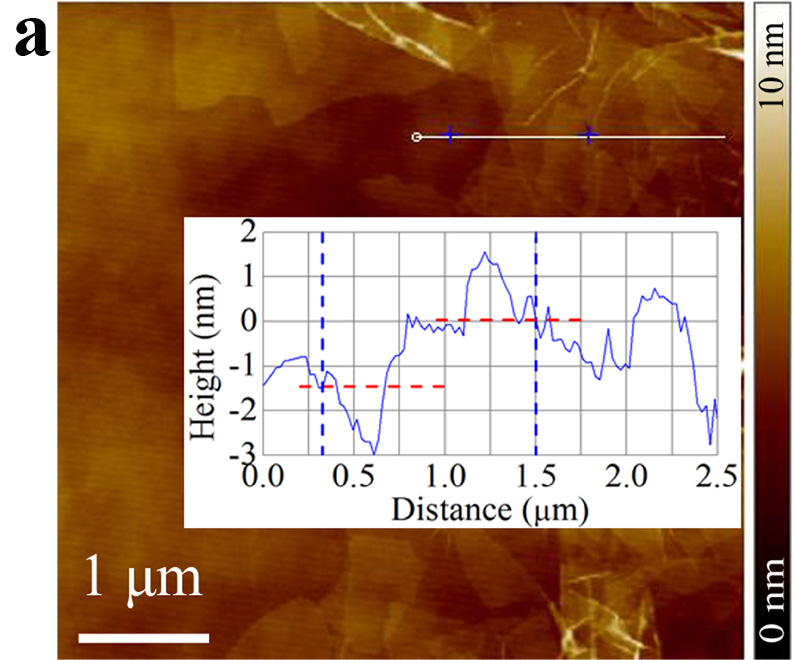


**Supplementary Figure S2 | Microstructure characterizations of aGO.** (**a**) AFM image and (**b**) Raman spectra. The black line indicates the Raman spectrum of an ultra-thin aGO sheet which thickness (~ 1nm) was predetermined by AFM scanning, and 1—5 are measurements of other five independent aGO sheets which are randomly selected after depositing aGO water solution onto SiO_2_/Si substrate. In Fig. S2a, One can find that, the aGO sheets are sized µm scale. Shown in the inset is the height profile corresponding to the white line with blue crosses. The blue dash lines correspond to the white blue crosses. The red dash lines indicate the thickness of two overlapped aGO sheets is ~ 1.4 nm, implying the aGO sheets are mostly monolayered ~ 0.7 nm. The Raman spectra of the ultra-thin aGO sheets (Fig. S2b) show the two prominent peaks at ca. 1350 and 1603 cm^-1^ corresponding to D and G bands, respectively. The 2D peak turns into two weak and wide “bumps” between ~ 2500 and ~ 3100 cm^-1^, similar to the Raman spectra of monolayered GO reported elsewhere^S1,S2^. Obviously, it opposed to the sharp and strong 2D peak seen in mechanically exfoliated graphene. By randomly selecting aGO sheets for Raman imaging (*eg.* Fig. S2b 1—4), we found that, most of them have the equal D/G ratio and the peaks appear at the same places. Actually, by normalizing their G peak, most measured signals are nearly coincided with which of the monolayered one. Furthermore, it is found that only few signals obviously deviate (*eg.* Fig. S2b 5). Therefore, the Raman spectra indicate further that most of our aGO sheets are monolayered.

**Supplementary Figure S3 | The typical fine-scanned O 1s XPS spectra of the OHG samples obtained at different annealing temperatures.** (**a**) OHG-700 and (**b**) OHG-900. The three main peaks around 530.88 ± 0.2, 531.83 ± 0.2, and 533.38 ± 0.2 eV were assigned to carbonyl (C=O), epoxy (aliphatic C–O), OH (hydroxyl C–O). One can find that OH peak is the dominant one despite that the measured O signals of the two samples fluctuated vigorously.

**Supplementary Figure S4 |** (**a**) The fine-scanned C 1s XPS spectra of aGO and the OHG samples. (**b**) Relative intensity to C–C *sp*^2^ peak area of oxygen groups. The peaks at 284.43 ± 0.2, 285.9 ± 0.2, 286.6 ± 0.2, and 287.5 ± 0.2 eV were assigned to C–C sp^2^ bonding, C–OH bonding (OH), C–O–C bonding (epoxy) and >C=O bonding (carbonyl), respectively. Note that, the peak at around 288.9 eV corresponding to COOH group (O=C–O, carboxyl) did not completely vanish even at 900 °C since carboxyl group was considered easy to be removed. We denoted the involved C=O signal responsible to this (ref. 22). The π→π* shakeup satellite peak around ~ 290.6 eV was also found in OHG samples, implying aromatic structure was to some extent been restored (refs 33-35). The relative intensities normalized to C–C sp^2^ peak area of oxygen groups were summarized in Fig. S4b. After annealing of aGO, it is found that (i) the ratios of both epoxy and carboxyl groups rapidly deceased to a minimum value, only tiny fluctuations could be seen; and (ii) the ratio of C–OH bonding in OHG is incontrovertibly the dominant one. Interestingly, according to the value ~ 15% of C–OH/C–C to calculate the O content in OHG samples (such as OHG-700), a higher O content (~ 13 at.%) will be obtained than actual results (~ 5 at.%), in agreement with other experiments (refs. 33 & 34). The phenomena can be assigned to the existent amorphous carbon or the disruption of *sp^2^* domain of graphene lattice by oxygen groups or other defects (ref. 36). As a result, the calculated peak area ratio of C–C *sp^2^* by deconvoluting the C 1s fine-scanned XPS spectra is always lower than the actual C ratio. Thus, by deconvoluting C 1s spectra, one can obtain a higher content of oxygen groups than the real one. This is the reason that we chose the contents of oxygen groups by deconvoluting O 1s spectra as the real content of oxygen groups in the main text.

**Supplementary Figure S5 | The measured magnetic signals of aGO with background by using 48.09 mg sample at** (**a**) 300 K and (**b**) 2 K**.** The solid black line is the guide for the eye only.

**Supplementary Figure S6 | Comparison of paramagnetic responses of the OHG and OH-imported OHG samples.** Mass magnetization dependences on applied magnetic field (*M – H*) measured at 2 K of (**a**) OHG-600 and OH-imported OHG-600, (**b**) OHG-500 and OH-imported OHG-500. Colorful symbols are the measurements and solid lines are fit to Brillouin function with *g* = 2. *S* is the fitted spin angular momentum number. OH-imported OHG was prepared according to ref. 48. In short, OHG (50 mg) was dispersed in NaOH aquous solution (40 ml, 2 M) with the aid of ultrasonication of 30 min. Next, the solution was transferred into a stainless steel reaction autoclave (50 ml) with a polytetrafluoroethylene linear, heated to 180 °C for 2h. After cooled to room temperature, the mixture was washed with deionized water for 20 times until the PH reached 7.0. Lastly, the mixture was dried in a vacuum oven at 50 °C. All the samples are paramagnetic by performing *M – T* measurements (not shown).

**Supplementary Table S1 | Comparison of magnetic properties of other graphene and its** derivatives.

| graphene and its derivatives | saturated magnetization (*M_s_*) | spin density | references |
| --- | --- | --- | --- |
| OHG-500 | 2.41 emu/g | 6 *μ_B_* / 1000 C | this study |
| graphene laminate | ~ 0.1 emu/g | 1 *μ_B_* / 20000 C | *Phys. Rev. Lett.* **105**, 207205 (2010) |
| fluorinated graphene | ~ 0.2 emu/g | 1 *μ_B_* / 1000 C | *Nat. Phys.* **8**, 199 (2012) |
| fluorinated graphene | ~ 0.8 emu/g | 3 *μ_B_* / 1000 C | *ACS Nano* **7***,* 6729 (2013) |
| H-doped graphene | 0.006 emu/g | Not mentioned | *ACS Nano* **7***,* 5930 (2013) |
| H-doped graphene | Not mentioned | Not mentioned | *Appl. Phys. Lett.* **98**, 193113 (2011) |
| C^4+^ / H^+^-irridiated graphene | < 0.02 emu/g | Not mentioned | *Nat. Phy.* **8**, 199 (2012) |
| N-irridiated graphene | Not mentioned | Not mentioned | *Appl. Phys. Lett.* **99**, 102504 (2011) |
| unzipped graphene nanoribbon | ~ 0.25 emu/g | Not mentioned | *Nano Lett.* **12**, 1210 (2012) |
| reduced graphene oxide | 0.02 emu/g | Not mentioned | *Nano Lett.* **9**, 220 (2009) |

**Supplementary Table S2 | The contents of the metal impurities of aGO and the OHG samples measured by ICP spectrometry.** The unit is ‘ppm’, and ‘ND’ denotes ‘not found’.

| samples | aGO | OHG-200 | OHG-300 | OHG-400 | OHG-500 | OHG-600 | OHG-700 | OHG-800 | OHG-900 |
| --- | --- | --- | --- | --- | --- | --- | --- | --- | --- |
| Fe | 0.4 | 1.6 | ND | 0.6 | 13 | ND | ND | 5.6 | 23.2 |
| Mn | 13.7 | 3.9 | 20.4 | 2.6 | 6.9 | 15.3 | 25.8 | 14.7 | 6.7 |
| Ni | ND | ND | ND | ND | ND | ND | ND | ND | ND |
| Co | ND | ND | ND | ND | ND | ND | ND | ND | ND |
| Cr | ND | ND | 1.1 | ND | ND | ND | 0.5 | ND | ND |
| Zn | ND | 7.5 | ND | ND | ND | ND | 12.1 | ND | ND |

**Supplementary References.**

S1. Jung, I., Dikin, D. A., Piner, R. D. & Ruoff, R. S. Tunable Electrical Conductivity of Individual Graphene Oxide Sheets Reduced at "Low" Temperatures. *Nano Lett.* **8**, 4283-4287 (2008).

S2. Eda, G. & Chhowalla, M. Chemically Derived Graphene Oxide: Towards Large-Area Thin-Film Electronics and Optoelectronics. *Adv. Mater.* **22**, 2392-2415 (2010).
